# COVID-19 symptoms are reduced by targeted hydration of the nose, larynx and trachea

**DOI:** 10.1038/s41598-022-08609-y

**Published:** 2022-03-29

**Authors:** Carolin Elizabeth George, Gerhard Scheuch, Ulf Seifart, Leeberk Raja Inbaraj, Sindhulina Chandrasingh, Indu K. Nair, Anthony J. Hickey, Michael R. Barer, Eve Fletcher, Rachel D. Field, Jonathan Salzman, Nathan Moelis, Dennis Ausiello, David A. Edwards

**Affiliations:** 1 Bangalore Baptist Hospital, Bangalore, India; 2 GS BIO-INHALATION GmbH, Germunden, Germany; 3 Klinik Sonnenblick, Marburg, Germany; 4 RTI International, Research Triangle Park, NC USA; 5 Respiratory Sciences, University of Leicester, Leicester, UK; 6 School of Engineering & Applied Sciences, Columbia University, New York, NY USA; 7 Sensory Cloud, 650 East Kendall St, Cambridge, MA USA; 8 School of Bioengineering, Northeastern University, Huntington Avenue, Boston, MA USA; 9 Massachusetts General Hospital, Boston, MA USA; 10 John A Paulson School of Engineering & Applied Sciences, Harvard University, Cambridge, MA USA

**Keywords:** Biophysics, Diseases, Health care

## Abstract

Dehydration of the upper airways increases risks of respiratory diseases from COVID-19 to asthma and COPD. We find in human volunteer studies involving 464 human subjects in Germany, the US, and India that respiratory droplet generation increases by up to 4 orders of magnitude in dehydration-associated states of advanced age (n = 357), elevated BMI-age (n = 148), strenuous exercise (n = 20) and SARS-CoV-2 infection (n = 87), and falls with hydration of the nose, larynx and trachea by calcium-rich hypertonic salts. We also find in a protocol of exercise-induced airway dehydration that hydration of the airways by calcium-rich salts increases oxygenation relative to a non-treatment control (P < 0.05). In a random control study of COVID-19 positive subjects (n = 40), thrice-a-day delivery of the calcium-rich hypertonic salts (active) suppressed respiratory droplet generation by 51% ± 11% and increased oxygen saturation over three days of treatment by 48.08% ± 9.61% (P < 0.001), while no changes were observed in the nasal-saline control group. Self-reported symptoms significantly declined in the active group and did not decline in the control group. Hydration of the upper airways appears promising as a non-drug approach for reducing risks of respiratory diseases such as COVID-19.

## Introduction

COVID-19 continues to have a devastating impact on human health^1^, economic development^2^ and wellbeing^3,4^. While the rapid and effective development of vaccines^5^ and drugs^6^ have reduced the burden of COVID-19 in many high and middle income regions, lack of access to or practical delivery of pharmaceutical solutions in low-income countries has created a growing gap in health, education, and economic prospects between the most and least wealthy people on the planet^7^. Dirty air and poor access to healthcare already threaten the lives of billions of people in low-income regions of the world^8^, where respiratory disease is the leading cause of death^8^. There is therefore an urgent need for practical, implementable and effective scientific solutions to minimize risks of respiratory disease such as COVID-19 among the least fortunate.

Getting moisture to the upper airways may be such a solution^9–12^.

Whole-body dehydration frequently accompanies COVID-19^12^ and is a potential common underlying factor in phenotypical states such as advanced age^13^ and high BMI^14^ associated with heightened risk of disease. Chronic sub-optimal systemic hydration appears to promote the presence of angiotensin converting enzyme 2 (ACE2) receptors in the lung, and increase capillary leakage of airway lining fluid^15^, among other systemic biochemical consequences that increase risks of COVID-19^12,15^.

Systemic dehydration also depletes water in those upper airway regions of the lungs responsible for hydrating inhaled air^16^, notably the nose, trachea and main bronchi, where ACE2 receptors have been found to be of particularly high density^17^. The lungs emit approximately 25% of daily total water mass loss in the process of hydrating inhaled air^18^. This loss derives from a combination of water evaporation from upper-airway mucus on inspiration, and from exhalation of moist air from the lungs on expiration^16^.

Alternating low and high humidity in the upper airways during tidal breathing creates a cyclical pattern of dehydration and rehydration that, when accompanied by systemic dehydration or the chronic breathing of dry air, can promote extreme thinning of upper airway lining fluid^11^, reduce cilia beat frequency^10^, and damage epithelial cells^19^. These and other effects^20^ of airway dehydration reduce the ability of the upper airways to clear inhaled contaminants filtered out of the air by the upper airways^21^ and harm natural function to protect the gas exchange regions of the lungs. Chronic dehydration of the upper airways therefore exacerbates allergies^22^, asthma^23^, COPD^11^ and airborne infections including influenza^24^ and COVID-19^25^.

**Figure 1:**
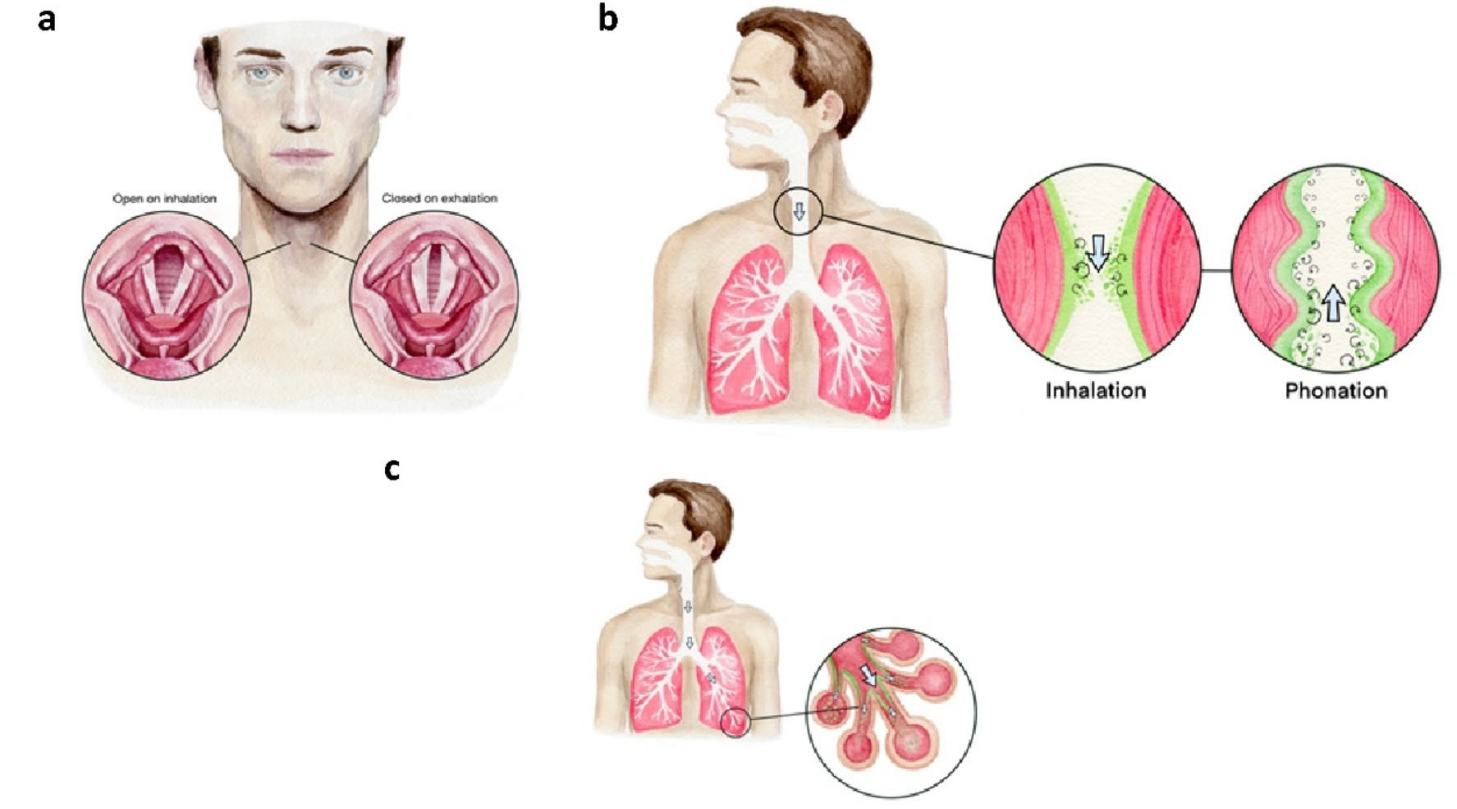
Artist renderings of the upper airways under normal and dehydrated conditions. (**A**) The human glottis during open (inhalation) and closed (exhalation) normal tidal breathing; (**B**) Airflow, droplet generation and turbulent eddies in the human larynx on normal inhalation; (**C**) Airflow, droplet generation and turbulent eddies on speech (phonation with exhalation).

Upper airway dehydration further alters the dynamics of the glottis^26^, the triangular-shaped narrow passage within the larynx bounded by the vocal folds (Fig. 1A). Responsible for sound generation, vocal folds are multi-layer tissues coated by mucus and epithelial cell layers^27^ that vibrate at around 100 Hz when exposed to pressures that exceed a threshold phonation pressure^28^ and to a degree shaped by viscoelastic properties that are highly water-dependent^29^. The glottis aperture fluctuates at around 1 Hz during normal tidal breathing, expanding on inhalation and contracting on exhalation^30^ by one to three-fold^31^. Dehydration of the glottis reduces flexibility of the vocal folds^32^, and can reduce glottal aperture and air flow as a consequence of reduced glottal pressure associated with diminished turbulent two-phase (droplet in air) mixing^33,34^.

Within the small aperture of the glottis, air flow conditions on normal breathing are characterized by temporal-spatial variations of laminar, transitional and fully turbulent conditions, with mean Reynolds numbers in the range of 1200–2400 and peak Reynolds numbers at the high shear region of the laryngeal jet of air that forms within the glottis of around 8000^35^. Recirculating eddies grow in size within the trachea on normal inhalation (air flow in the range of 15L/min to 30 L/min) from the larynx to the carina after which they rapidly dissipate^36^, generating surface waves and droplet breakup when Reynolds numbers exceed approximately 5000^37^. The laryngeal jet of air consequently drives upper airway respiratory droplet generation on inhalation^38^, and on exhalation^39^ with phonation (Fig. 1B).

Droplets generated in the upper airways on inhalation travel toward the periphery of the lungs, depositing throughout the airways as a function of droplet size. Reversing direction on exhalation, the smallest droplets originating in the upper airways exit the airways while mixing with droplets generated in the lung periphery by the temporary closing off of the small airways (Fig. 1C), a phenomenon augmented by residual volume (maximal inhalation and exhalation) breathing^40^, as with strenuous physical exercise.

The breathing of dry air has recently been observed to amplify upper-airway respiratory droplet generation^41^ on normal tidal breathing. Hydration of the upper airways by the breathing of humid air, the wearing of a face mask, or the direct delivery of isotonic or hypertonic saline droplets targeted to the posterior of the nose, larynx and trachea^42^ with mean droplet sizes around 8–12 μm reduces respiratory droplet generation to similar degrees and durations^41^. Beyond the consequences of hydration on glottal aperture and laryngeal-jet features—hydration of the larynx increases glottal aperture and reduces phonation threshold pressure^43^, the fluid mechanical basis of laryngeal hydration protocols for singers^43^—hydration alters respiratory droplet formation by volume expansion of the airway lining fluid. Volume expansion lowers surface active material concentration in airway lining fluid, altering mucus surface elasticity (propensity of the airway lining mucus surface to breakup), a phenomenon that has been observed in human^44^ and in vitro^45^ studies. The topical administration of divalent salts, notably calcium and magnesium chloride, further stabilizes mucus surfaces for prolonged periods of time^41^ by charge-association with anionic surfactants and mucin molecules^46^ proximate to the air–water interface. Hydration of the upper airways by the delivery of hypertonic divalent salts therefore reduces respiratory droplet generation in the upper airways to 4–6 h^41,47–49^ relative to a suppression of exhaled respiratory droplets of 60 to 90 min on the breathing of humid air or the delivery of normal saline droplets^41^.

We hypothesized that daily targeting of hypertonic divalent cation salt solutions to the upper airways would be an effective non-drug strategy to reduce health risks of COVID-19 by: (a) hydrating the upper airways in the early stages of SARS-CoV-2 infection to improve upper-airway clearance and; (b) reducing respiratory droplet generation and promoting oxygenation otherwise diminished by dehydration of the larynx. We studied respiratory droplet generation and oxygenation in a range of phenotypical states, including those characterized by whole-body dehydration, and following upper airway hydration. We then examined the treatment effects of upper airway hydration in moderately symptomatic COVID-19 patients in a randomized control clinical study. Our findings are reported here.

## Results

### Exhaled aerosol variation in states of whole-body dehydration

**Figure 2:**
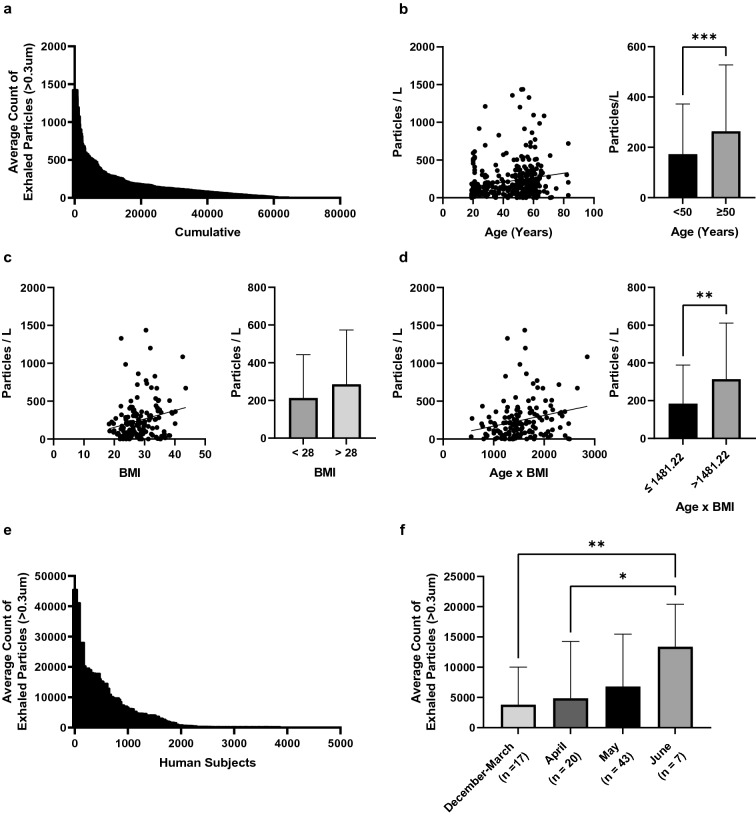
Exhaled aerosol particle numbers among healthy and infected human subjects in Germany and India following normal tidal breathing. (**A**) Exhaled aerosol particle numbers for 357 healthy human subjects in Marburg Germany. (**B**) Exhaled aerosol particle numbers versus age, with median = 50 years. (**C**) Exhaled aerosol particle numbers versus BMI, with median = 28. (**D**) Exhaled aerosol particle numbers versus BMI-age, with median = 1481.22. (**E**) Exhaled aerosol particle numbers from 87 mildly symptomatic COVID-19 subjects. (**F**) Mean exhaled aerosol numbers for the 87 COVID-19 patients as a function of time period of infection and recruitment. Error bars represent standard errors of the mean. *P < 0.05, **P < 0.005, ***P < 0.0005.

To assess natural variations in respiratory droplet generation among healthy human subjects, we measured exhaled aerosol in 357 volunteers in Marburg, Germany at 20–25 C and 20–40% relative humidity. Exhaled aerosol numbers with normal tidal breathing (Fig. 2A) ranged from below detection limit (1–2 particles per liter of air) to over 1000 thousand particles per liter of air. Exhaled aerosol droplets were predominately submicron (see Supplemental Material). No significant difference in exhaled aerosol or size distribution was observed between male (145) and female (212) subjects (P > 0.05) or between (self-identified) smoker (62) and non-smoker (195) subjects (P > 0.05) (Supplemental Material). Exhaled aerosol numbers did increase significantly (P = 0.0002) with age (n = 357) (Fig. 2B) and trended higher (P = 0.057) with BMI (n = 157) (Fig. 2C). Exhaled aerosol numbers significantly correlated (P = 0.0031) with advanced BMI-age (Fig. 2D) (n = 157).

We also measured exhaled aerosol in 87 mildly symptomatic COVID-19 positive patients admitted into Bangalore Baptist Hospital between December 2020 and June 2021 as shown in Fig. 2E. Exhaled aerosol numbers grew by 323% (P = 0.0031) among the 87 subjects (Fig. 2F) from the first cases in December-March to the last in early June 2021 as the delta variant (B.1.617) spread from first detection in India in late 2020 to over 60% of all cases in late May 2021^50^. Mean exhaled aerosol droplet numbers for the infected subjects (Fig. 2E) were significantly more numerous (mean 6,300 ± 1,792 particles per liter) than with the non-infected subjects (Fig. 2A) (mean 214 ± 31.3 particles per liter). No significant differences were observed in submicron size fraction between male (93.4% ± 6.8%) and female (92.2% ± 6.6%) subjects. Mean exhaled aerosol among those with normal C-Reactive Protein (CRP) levels (less than 10 mg/L) was not significantly different (P = 0.362) than mean exhaled aerosol of those with elevated CRP levels (greater than 10 mg/L). We identified SARS-CoV-2 RNA in several exhaled breath samples as further described in the Supplemental Material.

### Exhaled aerosol reduction by hydrating dry airways with calcium-rich hypertonic salts

Given the recent finding that topical airway dehydration amplifies exhaled aerosol^41^, and delivery of calcium-rich hypertonic saline to the upper airways diminishes exhaled aerosol for several hours^41,47–49^, we sought to explore the interrelated roles of systemic and topical (calcium-enriched hypertonic saline) hydration on exhaled aerosol during normal tidal breathing in a randomized two-armed interventional study of exercise-induced dehydration in 20 young (22–45 years of age), low-BMI human volunteers in Boston, Massachusetts.

**Figure 3:**
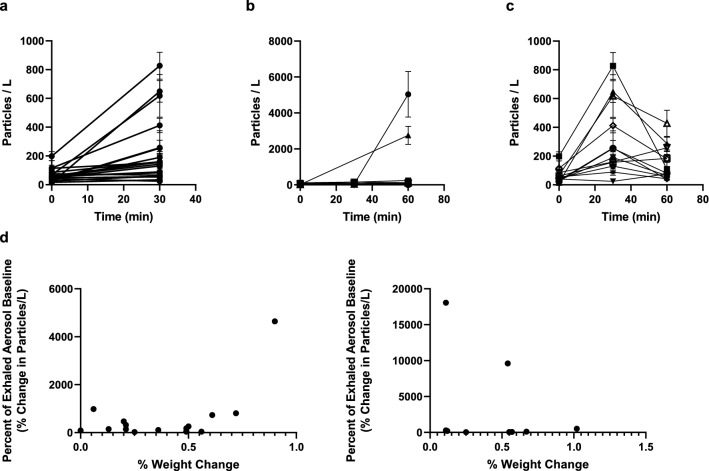
Exhaled aerosol particle numbers from healthy human subjects in an exercise-induced dehydration study. Shown are (**A**) all subjects before and after 30 min of exercise; (**B**) all non-treatment control subjects before, during and after 60 min of exercise; (**C**) all active subjects before and after administration at 30 min of exercise of upper-airway salts; (**D**) all subjects as a function of dehydration weight loss over the course of the exercise-induced dehydration study after 30 min (left) and all control subjects after 60 min (right). Error bars represent standard errors of the mean.

Healthy volunteers (17 males, 3 females, no smokers) participated in a coordinated workout that involved weight training and other physical exercises over 60 min within an air-conditioned gymnasium at 20–25 C and 50–70% relative humidity with all exhaled aerosol measurements performed once subjects had recovered normal tidal breathing. Water loss among the participants ranged from 0.1% to 0.9% of total body mass over the course of the 60 min. At 30 min into the workout half of the subjects were randomly selected to receive by nasal inhalation a calcium-rich hypertonic salt solution (4.99% calcium chloride and 0.01% sodium chloride by weight) with mean droplet diameter around 10 μm (approximately 20% of the droplets smaller than 7 μm) targeting the nose, trachea, and main bronchi (see Supplemental Material). Exhaled aerosol numbers (Fig. 3A) increased significantly for all subjects (P = 0.002) following 30 min of exercise from mean values of 58.8 ± 17.1 particles per liter of air (n = 21) to 220.6 ± 97.5 particles per liter of air (n = 19). With the continuation of exercise to 60 min for the control group mean exhaled aerosol trended higher (855 ± 995 particles per liter of air) while without significance relative to the exhaled aerosol levels at 30 min (P = 0.204). For the active group at 60 min (30 min post-delivery of the calcium-rich hypertonic salts) exhaled aerosol significantly diminished (P = 0.045) with mean exhaled aerosol of 151.9 ± 113.2 particles per liter of air (Fig. 3C). Exhaled aerosol generally increased with weight loss for all subjects during the first 30 min of exercise (Fig. 3D, left). Over the subsequent 30 min of exercise (up to 60 min from start of exercise) exhaled aerosol increased dramatically for two of the control subjects (Fig. 3D, right), while correlation between total body mass loss and exhaled aerosol disappeared—for all subjects who received no topical hydration between 0 and 60 min exhaled aerosol increase relative to baseline above the median weight loss of 0.24% (688.3% ± 448.9%) was not significant (P = 0.33) relative to exhaled aerosol increase below the median weight loss (exhaled 225.4% ± 97.7%).

To further explore the interrelationship between systemic and topic hydration, we preliminarily evaluated pulse oxygen saturation pressure in a subset of the human volunteer subjects. Mean oxygen saturation fell significantly (P < 0.05) for all subjects (n = 6) from 98.7% ± 0.9% prior to exercise to 96.7% ± 0.5% at 30 min of exercise. Mean oxygen saturation for the control group (n = 3) reached 96.5% ± 0.8% at 60 min post the commencement of exercise, an insignificant change relative to levels at 30 min while significantly below the pre-exercise levels (P = 0.01). For those subjects (n = 3) who received the laryngeal-targeted calcium-rich salts at 30 min post exercise, oxygen saturation rose significantly (P < 0.05) for two of the three subjects and remained unchanged (P > 0.05) for the third, a trend previously reported on exercise-induced dehydration following systemic hydration^41^; mean oxygen saturation for the group was 97.1% ± 0.5% at 60 min post the commencement of exercise.

### Random control COVID-19 treatment study with calcium-rich hypertonic salts

Given these findings, and the dehydration that accompanies COVID-19^12^, we evaluated the effect of upper airway hydration on respiratory response to COVID-19 infection. We studied daily administration (thrice a day for three days) of calcium-rich hypertonic salts to the upper airways on exhaled aerosol, oxygen saturation, and disease symptoms in a randomized double-blinded nasal-saline control study of 40 moderately symptomatic COVID-19 patients admitted into Bangalore Baptist Hospital during the period May–June when the delta variant predominated infections in India^50^. Most of the COVID-19-positive subjects in the two arms of the study entered the hospital with fever, cough, body pain and loss of smell or taste sensation (Table 1), and mean initial self-reported symptom scores of 3.15 ± 0.17 (no statistical difference in symptom scores was observed between the two groups, P = 0.599). Three of the subjects in the active group were escalated to intensive care prior to completing the three days of treatment and were therefore excluded from the post-treatment results. See Table 1.

**Table 1:** Patient assessment data for the 40 COVID-19 positive human subjects in the randomized control study of daily nasal and upper airway administration of calcium-rich salts (FEND) at Bangalore Baptist Hospital.

Participant No	Group	Age	Gender (M/F)	Blood Group	CRP	D dimer	Symptomatic	Fever?	Cough?	Difficulty Breathing?	Smell or Taste Difference?	Diarrhea or Vomit?	Body Pain?	Temp. (F)	Sp02	Temp. (F) (2nd Reading)	Spo2 (2nd Reading)	Temp. (F) (3rd Reading)	Spo2 (3rd Reading)	Disease Severity Classification	Subjective Symptom Score Day 0	Day 1	Day 2	Day 3	Day 4	Day 5	IV Antibiotic?	Steroid?	Outcome
1	Active	30	F		6.4	271	A	No	Yes	No	No	No	Yes	98	96	98.2	96	98	98	Mild	3	2	2				No	No	After 2 days, she was discharged as became better
2	Active	16	F		1.1	215	S	Yes	Yes	No	Yes	No	Yes	98.1	98	98.3	99	98.3	99	Mild	3	3	2	2	2		No		Discharged
3	Control	42	M	O+	8.4	165	S	Y	Y	N	N	N	Y	100	96	98	97	98	98	Mild	3	3	2	2			N	N	Discomfort in conutinuing nasal spray; Discharged
4	Control	40	M	O+	27.2	253	S	N	Y	N	Y	N	N	99	97	98.3	96	98	97	Moderate	4	4	3	3	3	2	Y	Y	Discharged
5	Active	23	M	O+	2.8	218	S	Y	N	N	Y	N	Y	98.2	96	98.1	98	98.4	98	Mild	3	2	2	1	1		N		Discharged
6	Active	45	M	A+ve	6.4	250	S	Y	Y	N	Y	Y	Y	98	98	98.3	99	98	99	Mild	3	3	2	2			N	N	Discharged
7	Active	41	M	A+ve	31.3	304	S	Y	Y	Y	Y	N	Y	98.2	98	98	99	98	99	Mild	4	4	3	2	2		Y	N	Discharged
8	Control	28	F	O+	1.9	198	S	Y	Y	N	N	N	Y	98.3	99	98	98	98.4	99	Mild	3	3	2	2			N	N	Discharged
9	Control	39	F	O+	25.4	290	S	Y	Y	N	N	Y	Y	98	98	98.2	98	98.1	98	Mild	3	3	2				N	N	Discharged
10	Control	28	F	O+	2.3	197	S	Y	Y	N	N	N	Y	99	96	98	96	98	98	Mild	3	3	2				N	N	Discharged
11	Control	47	M	A+ve	23.1	218	S	N	Y	N	Y	N	N	98	97	98	96	98	98	Mild	3	3	3	3			N	N	Discharged
12	Control	41	M	A+ve	20.1	324	A	N	Y	N	N	N	Y	98.1	98	98.2	97	98.1	98	Mild	3	3	2				N	N	Discharged
13	Active	47	M		115.5	291	S	Y	Y	Y	N	Y	Y	98.1	97	97.8	96			Moderate	4	3	3				Y	Y	escalated/klebsiella infection, Ctseverity 10/25
14	Active	32	M	A+ve	11.5	248	S	Y	Y	N	N	Y	Y	98	97	98	98	98.1	99	Mild	3	2	2				N	N	Discharged
15	Active	20	M	O+ve	17.7	324	S	Y	Y	N	Y	N	Y	98	96	98.3	98	98	98	Mild	2	2	2	1			N	N	Discharged
16	Active	42	M	O+ve	35.2	409	S	N	Y	N	N	Y	N	98.2	95	98	96	98.1	98	Mild	3	3	3	2	2	2	Y	Y	Discharged
17	Active	38	M	O+ve	28.8	229	S	Y	Y	Y	N	N	Y	98.1	97	98	93			Moderate	4	4	4				Y	Y	escalated
18	Control	45	F	O+ve	7.9	244	S	Y	Y	Y	N	N	Y	98	96	98.1	96	98	96	Mild	3	3	2	2	2		Y	N	escalated to increased d dimer
19	Control	53	F	B+ve	5.2	187	S	Y	Y	N	N	Y	Y	98	97	97.8	96	98.3	97	Mild	3	3	3	3	3	2	Y	Y	steroids were given for 5 days
20	Control	54	F	–	13	163	S	Y	N	N	N	N	Y	99	97	98	98	98	99	Mild	2	2	2	2			Y	Y	Discharged
21	Control	44	F	O−ve	13.6	208	S	Y	Y	Y	N	Y	Y	98.2	98	98.5	97	98.4	98	Mild	3	3	2	3	2		Y	N	Diccharged -10/05/2021
22	Active	37	M	A+	3.2	201	S	Y	N	N	Y	N	Y	99	97	99	95	98	98	Moderate	3	3	3				N	Y	Shifted to W-6
23	Active	45	M	A+	214.5	357	S	Y	Y	N	N	Y	Y	98	97	98.8	95	98	93	Moderate	3	3					Y	Y	Shited to ER on 8/05/2021
24	Active	24	M	O+ve	6.4	179	A	Y	Y	N	N	N	Y	98	97	98.1	98			Mild	3	1	1				N	N	Discharged
25	Active	41	M	–	49.7	187	S	N	Y	Y	N	N	Y	98	94	98.4	95	98.1	98	Moderate	2	2	1	1			N	N	Discharged -8/05/2021
26	Active	29	M	A+ve	9	98	S	N	N	N	N	Y	Y	98.2	97	98.4	99	98.1	99	mild	3	3	3	2	2		Y	N	Discharged -11/05/2021
27	Control	53	F	O+ve	95.9	187	S	Y	Y	N	Y	N	Y	98	95	98.2	95	98.1	97	moderate	3	3	3	3	2		Y	Y	Discharged
28	Control	57	F		2.8	290	S	Y	Y	Y	Y	Y	Y	98	96	97.9	98	98.3	96	Mild	3	3	2	2			N	N	Discharged -10/05/2021
29	Active	55	M	B+ve	66.5	220	S	Y	Y	N	Y	N	Y	98	96	98.2	99			Mild	3	2	1				N	Y (oral 1 dose)	Discharged -11/05/2021
30	Active	33	M	O−ve	5.7	170	S	Y	Y	Y	Y	Y	N	98	97	98	99	98	99	Mild	3	2	2	1			N	N	Discharged-14/05/2021
31	Control	33	M	A+ve	85.2	189	S	Y	Y	Y	Y	Y	Y	98	97	98.1	97	98.2	96	Mild	4	3	3	3			N	Y	Discharged
32	Control	50	F	–	12.52	204	S	Y	Y	N	N	Y	Y	98.5	96	98.4	98	98.1	97	Mild	3	2	2				N	N	Discharged-
33	Control	44	F	–	3.67	213	S	Y	Y	N	N	Y	Y	98	98	98.5	98	98	98	Mild	3	2	2				N	N	Discharged
34	Control	33	F				A	Y	N	N	Y	Y	Y							Mild	3	3	2				N		Discharged 14/06/2021
35	Control	48	M	A+ve	0.7	224	S	Y	Y	N	N	N	Y	98	99	98	98	98	99	Mild	3	3	3	2			N		Discharged 17/06/2021
36	Control	30	M	0+ve	31.1	234	S	Y	Y	N	Y	N	N	98	98	98.1	95	98	95	Mild	4	4	3	3			Y		Discharged
37	Control	29	M	B+ve	34.8	221	S	N	Y	Y	Y	N	N	100.1	95	97.1	95	97	94	Mild	4	4	2	2			Y		Discharged 16/06/2021
38	Active	46	M	A+ve	45.4	324	S	Y	Y	N	Y	N	Y	98.6	96	101	98	97	98	Mild	4	4	2	2			N		Discharged
39	Active	29	F	B+ve	1.19	261	S	Y	Y	Y	Y	Y	Y	99.6	95	97.6	99	97	99	Mild	4	4	1	1			N		Discharged
40	Active	42	M	A+ve	23.7	403	S	Y	Y	N	N	N	N	100.1	97	98	98	97	98	Mild	3	3	1	1			N		Discharged

See Supplemental Material for X-Ray and exhaled aerosol data.

**Figure 4:**
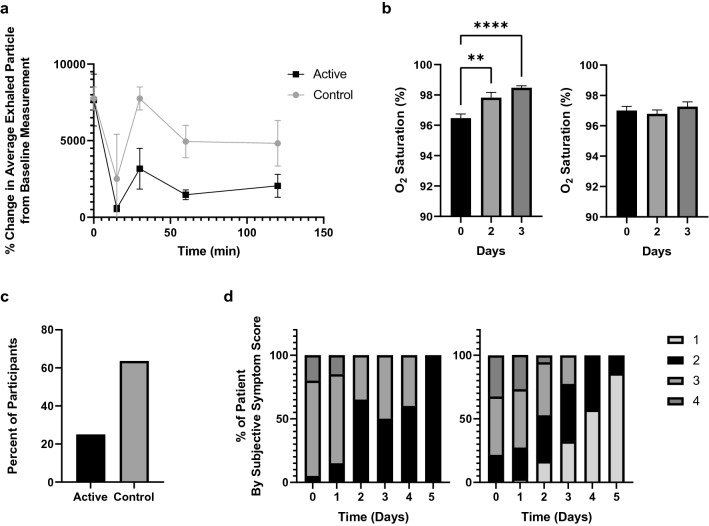
Drug intervention and COVID-19 symptom measures following administration of FEND or the Simply Saline control. (**A**) Exhaled aerosol particle numbers from 39 mildly symptomatic COVID-19 subjects following administration of hypertonic calcium-rich salts targeting the upper airways (active—FEND), and 39 mildly symptomatic COVID-19 subjects following administration of nasal saline spray (Simply Saline—control); (**B**) Oxygen saturation levels as a function of days of administration of FEND (left, n = 17)) and Simply Saline (right, n = 20) control; (**C**) % intravenous antibiotic or steroid intervention required among those subjects with high inflammation in the FEND cohort (9 of 20 subjects) and in the Simply Saline cohort (9 of 19 subjects); (**D**) Self-reported symptom scores (on scale 1 to 5, with 1 = no symptoms, and 5 = most severe symptoms) as a function of days of hospitalization and administration over the first three days of FEND (right) and Simply Saline control (left). Error bars represent standard errors of the mean. **P < 0.0025, ***P < 0.0001.

Administration of the active reduced exhaled aerosol in all subjects (Fig. 4A) 30 min post administration, and for all but one subject for the duration of the assessment (up to 2 h post administration). Administration of the Simply Saline control (Fig. 4B) did not change overall exhaled aerosol for the group (P = 0.122), while some subjects did exhale fewer respiratory droplets post administration of the nasal saline control, suggesting the possibility that some patients in the control arm of the study may have experienced an active effect of the nasal saline on aerosol generation in the trachea given prone positions in hospital beds and post-nasal drip, a possibility further discussed in the Supplemental Material.

Oxygen saturation levels began at similar levels for the active and control groups at the start of the study (Fig. 4B). Over the course of the three days of administration, oxygen saturation rose significantly (P = 5.981e−07) in the active group and did not change (P = 0.533) in the Simply Saline control group (Fig. 4B). Daily reduction of self-reported symptom scores was observed in the active group from Day 2 with 86% of all subjects discharged without symptoms while 0% of the control group ended their stay in the hospital without symptoms (Fig. 4D). Among the subjects of each group who entered the trial with elevated CRP levels (CRP > 10 mg/mL), intravenous antibiotics or steroids were needed in only 25% of the active group (n = 8) versus 63.64% of the saline control group (n = 11) (Fig. 4C). CRP levels fell from day 1 to day 3 in 75% of the active group and in 52.6% of the control group (Table 1).

We further monitored exhaled aerosol, symptoms scores and oxygen saturation levels for 10 of the 37 non-treated subjects over the course of their hospitalization as reported in the Supplemental Material. Oxygen saturation levels and treatment scores remained unchanged from admission to release from the hospital (see Supplemental Material).

## Discussion

Poor hydration has myriad adverse effects on the human body’s ability to resist infection and appears to be a common underlying factor in phenotypical states at high risk of COVID-19^12,15^. Our findings across study sites in the US, Germany and India appear to be the first to specifically implicate laryngeal and tracheal dehydration as a factor in the susceptibility to and worsening of symptoms in COVID-19.

Consistent with a recent report^51^, we found that exhaled aerosol is highest in the elderly (Fig. 2B), the obese (Fig. 2C,D), and those infected by SARS-CoV-2 (Fig. 2E), relative to young and healthy (low BMI, non-infected) human subjects. We also find that exhaled aerosol is amplified in young and healthy human subjects following exercise-induced whole-body dehydration—reaching levels (Fig. 3A,B) otherwise observed in the non-infected elderly and obese human subjects (Fig. 2B,C). Topical hydration of young and healthy (exercise-induced dehydrated) subjects with salt droplets sized (over 80% of the droplets larger than 7 μm) to mostly deposit above the carina diminishes exhaled aerosol to normal low levels even while the subjects remain in a whole-body dehydrated state (Fig. 3C). These findings are consistent with recent findings^41^ that healthy human subjects on moving from a dry air environment to a humid environment exhaled significantly fewer respiratory droplets. The central and lower airways being insensitive to the humidity of the environment, our results suggest that the breathing of humid air reduces dehydration of the upper airways, providing upper airway hydration similar to what we observe following hypertonic salt delivery in our exercise-induced dehydration study (Fig. 3C). Indeed, in previous work^41^ we find that delivery of isotonic saline or hypertonic saline with 8–12 μm mean-diameter salt droplets diminishes exhaled aerosol to levels equivalent to the breathing of humid air—consistent with what we observe in the present study.

Laryngeal respiratory droplet generation appears to be driven by the fact that on normal inhalation air passing through the larynx accelerates in the vicinity of the glottis (Fig. 1B) to attain turbulent flow conditions (Re up to 8000) at the center of a laryngeal jet of air that exits the glottis and enters the trachea^34,35^. This jet of air shears airway surface water lining the glottis, trachea and main bronchi closest to the carina threatening surface instability when Reynolds numbers exceed around 5000^36^. When the airway lining fluid is depleted either by the breathing of dry air or by systemic dehydration, its volume diminishes, salt concentrations increase, and surfactant concentration on mucus airway lining fluid increases. Enhanced surfactant concentration on airway lining fluid destabilizes surfaces and promotes droplet breakup^43,44^. On the other hand, delivery of hypertonic salt droplets to the trachea and main bronchi increases water content both by the delivery of water mass and the hypertonicity of the water delivered. Our recent work^41,46–48^ indicates that when the salts are divalent they prolong the stabilization effect of the mucus surface for 4–6 h relative to 1–2 h on the breathing of humid air or the delivery of sodium chloride salt to the same anatomical regions.

Analysis of respiratory droplet composition has revealed the presence of lung surfactant and the absence of mucin, implicating the smaller airways as a more probable site of generation than the upper airways^52^. However, mucus exists in the upper airways as a hydrogel^53^. Evaporating hydrogels naturally develop a thin film of water over free air surfaces in the process of seeking equilibrium with moisture in the air^54^. In the first few minutes of evaporation, hydrogels therefore tend to evaporate at a rate that is indistinguishable from water itself—while with the thin water film disappearing, evaporation rate precipitously declines^55^. In the upper airways, evaporation occurs from mucus surfaces on inhalation, hydrating inhaled air, while on exhalation highly humid air from the central and lower airways passes over upper airway mucus surfaces. The ability of the lungs to properly hydrate inhaled air prior to the penetration of air into the central and lower airways is contingent on the lungs releasing to the external environment approximately 1/2 L of water per day^16^. With the preponderance of airway lining fluid volume existing in the small airways and alveolar region of the lungs^16^, movement of airway lining fluid from the lower to the upper airways is essential in the form of condensate from the fully saturated air exhaled out of the lungs, as well as deposition of respiratory droplets generated in the small airways (Fig. 1C). While further research is needed to clarify the nature of the phenomenon, conceivably the combination of water film over the surface of upper airway mucus, transfer of airway lining fluid from the lower airways to the upper airways on exhalation, and unlikelihood of hydrogel (mucin) molecules aerosolizing under the shear forces that otherwise easily breakup water surfaces, contribute to respiratory droplets, wherever they form in the airways, tending to be of similar composition.

Small airway respiratory droplet generation also occurs^40,52^. In our study we find particularly that when subjects deeply exhale and inhale, often referred to as residual volume breathing, exhaled aerosol numbers are much higher than on normal tidal breathing (see Supplemental Material), and as these numbers are not appreciably diminished by upper airway delivery of hypertonic salts (Supplemental Material), these residual-volume-breathing respiratory droplets appear to originate largely in the smaller airways. Within the airways there then appears to be a traffic of respiratory droplets from the upper airways to the lower airways, and from the lower airways to the upper airways, this traffic predominating in one direction or the other depending on many factors that should be further explored, including the degree of air volume expired, the rate of air flow, and the health and hydration state of the individual. We particularly find in the present study that the respiratory droplet generation in and movement from upper to lower airways is detrimental to lower respiratory tract disease, and notably to COVID-19.

We observed a growth in exhaled respiratory droplets of 323% among the 87 patient volunteers (Fig. 1B) from December 2020 to June 2021 as the delta variant grew from a small minority of cases to greater than 60% of sequenced infections^50^. Differences in average age and disease severity of patients in our study (Table 1) were insignificant between December 2020 and June 2021, as were environmental factors including relative humidity and air quality, suggesting that increase in proportion of delta variant infections may be a cause of the amplification of respiratory droplet generation over the duration of our study and a factor in the increased contagiousness of the delta variant of SARS-CoV-2. In general, we find that respiratory droplet numbers are higher in those infected by SARS-CoV-2 (Fig. 2E) than non-infected individuals (Fig. 2A), consistent with our previous report^51^. Causes of this growth in exhaled aerosol with the advance of the delta variant infections may be multiple^56^, including variances in upper airway dehydration and variances in surface activity—respiratory droplet generation having been shown to increase with the addition of surfactant to airway lining mucus^44^, which increases propensity of airway lining mucus to break up^38^.

Our observations of reduced symptoms and need for intravenous antibiotic and steroid intervention with laryngeal and tracheal hydration suggest that upper-airway respiratory droplet generation may contribute to the worsening of symptoms of COVID-19 owing to progression of the virus deeper into the lungs by the breakup of airway lining fluid in the upper airways where SARS-CoV-2 infection generally begins. This hypothesis is at least consistent with the preponderance of data pointing to the role of respiratory droplets in the airborne transmission of COVID-19^57,58^. Diminution of respiratory droplet generation, and hydration of the glottis, by the delivery of upper airway hypertonic salts therefore reduces symptoms and improves oxygenation, both of which may contribute to a diminished need for intravenous drug intervention in those patients with high inflammation.

The impact of upper airway hydration on oxygenation needs further study. We find that oxygen saturation increases in moderately symptomatic COVID-19 patients over three days of daily administration of calcium-rich hypertonic salts (Fig. 4C), as well as during exercise-induced dehydration, although the latter conclusion is based on a small data set and will need to be validated by further study. Possibly glottal aperture, known to increase with increasing glottal hydration^33,34^, diminishes in COVID-19 and with exercise-induced dehydration. In processes of physical exercise and progression of COVID-19, upper airway dehydration may add to the effects of elevated release of oxygen by hemoglobin (in states of exercise) and impairment of oxygen absorption in the gas exchange regions of the lungs (in symptomatic disease), thus contributing to low oxygen saturation at least prior to when the latter may play a predominant role. Further research is obviously needed.

Mechanistically, future research should explore the impact of laryngeal hydration by (monovalent, divalent, isotonic and hypertonic) salts in laryngeal models as well as on oxygenation and respiratory droplet generation in the elderly, the obese, diabetics, athletes and those with an airborne infection. Clinically, confirmation of the principal findings of our study in asymptomatic and symptomatic COVID-19 patients should be pursued in larger patient cohorts with particular attention to accurately quantifying viral RNA in exhaled breath.

We believe the growing global respiratory health crisis makes it especially important to explore prophylactic, therapeutic and anti-contagion benefits of regular daily upper airway hygiene or hydration in populations at high risk of respiratory disease and in low-income environments where they are otherwise without access to proper hygiene (wearing of clean face masks, social distancing, and other modes of hygienic living including hand-washing), drugs and vaccination.

Divalent cation hypertonic salt delivery to the nose, larynx and trachea appears to be promising as a simple, safe, non-drug, daily hygiene strategy for upper airway hydration and facilitating natural clearance of inhaled contaminants, including SARS-CoV-2. A non-pathogen specific hygienic approach for respiratory disease could be an inexpensive and easily-adopted approach to supporting global respiratory health in the light of the ongoing pandemic and the worsening of air quality associated with climate change.

## Methods

**Figure 5:**
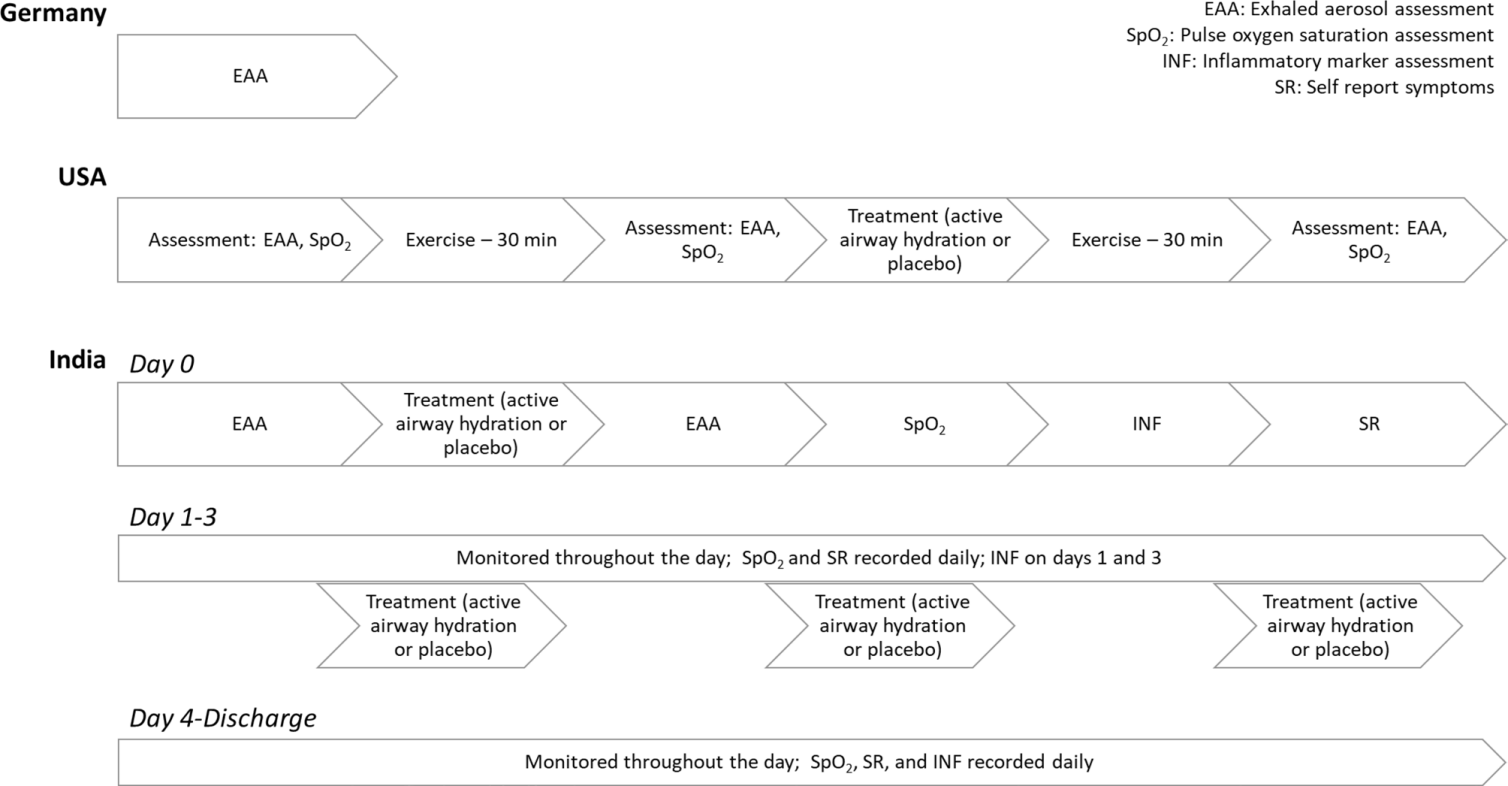
Flowchart of multisite human volunteer study.

All human studies (Fig. 5) at our three study sites (Bangalore, Marburg, Boston) were conducted in accordance with relevant regulations, and in accordance with the Declaration of Helsinki. Exclusion criteria included at all three sites included: human volunteers younger than 18 years or older than 70; negative (for Germany and US) or positive (India) nasopharyngeal swab for SARS-Cov-2 (RT-PCR) (within 1 week); subjects with chronic debilitating illness like cancer, immune deficiency etc.; and notably in at Bangalore Baptist Hospital severely ill subjects, subjects with mental illness/psychiatric medications, and subjects unable to complete 3 full days of treatment with active intervention or placebo.

### Germany study site: exhaled aerosol study recruitment and enrollment

We recruited 357 healthy human subject volunteers at the Klinik Sonnenblick, Marburg from January to March 2021. Of these there were 212 male and 145 female, 65 smokers and 292 non-smokers, ages 18–83, and BMI 17 to 43 (see Supplemental Material). We obtained IRB approval for the study from the Ethics Committee at the University Marburg. All participants provided written informed consent prior to enrollment. Age, weight, height, and smoking status was documented for all of the subjects and lung health parameters documented for a subset of 157 subjects (see Supplemental Material). Subjects exhaled aerosol at normal tidal breathing and their exhaled aerosol particle numbers were assessed as further described below.

### India study site: COVID-19 patient study recruitment and enrollment

We recruited 87 human subject volunteers ages 16–57 among mildly symptomatic COVID-19 patients at Bangalore Baptist Hospital (BBH) during a phase of the Indian pandemic (December to June 2021) over which sequenced BBH infections of the delta (B.1.617.2) coronavirus variant increased from a small minority of cases to greater than 60% of Indian infections^40^. Participants were screened for SARS CoV-2 infection by polymerase chain reaction (PCR) before enrollment. We obtained IRB approval for the study from the Ethics Committee at Bangalore Baptist Hospital. All participants provided written informed consent prior to enrollment. We assessed symptoms of all 87 participants immediately after enrollment by analysis of blood markers of inflammation (CRP, D-Dimer), lung X-Ray, pulse oximetry, temperature and self-reported symptoms (fever, cough, diarrhea, loss of taste/smell, breathing difficulty, body pain). Exhaled particles of all participants were measured by the particle detector method described below, and 40 were randomly assigned to two treatment cohorts (Table 1) with a block randomization design in which patients, clinical and research staff were all blinded to the use of the active or the control. We used a sequentially numbered, opaque, sealed envelope (SNOSE) technique in which the randomization group is written on a paper kept in an opaque sealed envelope, which is labeled serially. For each person recruited, the numbered envelope corresponding to that person's recruitment number is opened to see the allotment category. An active (FEND) cohort of 20 subjects received by nasal inhalation a calcium-rich hypertonic salt solution (4.72% CaCl_2_, 0.31% NaCl) via a hand-held, vibrating-mesh nebulizer (see Supplemental Material) and with a median volume droplet diameter of 8–12 μm designed to target the nose, trachea and main bronchi. A (Simply Saline) control cohort of 20 subjects received by nasal spray an isotonic saline (0.9% sodium chloride) with droplets sized to deposit in the nose of approximately 50 μm mean diameter. All participants received the active or the control three times a day over the first three days following the start of the study. Patients were blinded to whether they received the active or the control. We evaluated exhaled aerosol of all active and control patients before and after salt administration for one to two hours post-delivery. We monitored all patients each day for oxygen saturation levels, body temperature, IV antibiotic and steroid treatment (where needed), and self-reported symptoms on a scale of 1–5 (increasing in scale from no symptoms to the most severe symptoms). We used face-mask sampling to detect and quantify exhaled SARS-CoV-2 as further described in the Supplemental Material. We measured C-Reactive Protein (CRP) and D-Dimer in blood samples of all participants at the commencement of the study to assess inflammation and need for intravenous antibiotics. On the first day and each subsequent day we measured oxygen saturation by pulse oximetry. All participants in the India study received oral antibiotics (azithromycin) and were treated for fever with paracetamol. We responded to persistent fever or bad cough not relieved by symptomatic management by administering intravenous antibiotics. We administered steroid in response to persistent distressing symptoms, falling oxygen saturation (below 95%) or increased CRP or D Dimer. Failure to maintain adequate oxygen saturation levels or reduce distressing symptoms resulted in escalation to intensive care. We excluded results from the randomized control study of all subjects who escalated to intensive care before completing the three days of FEND or Simply Saline administration. We also excluded from the study those patients who required supplemental oxygen prior to admission or during the first three days post admission (blood oxygen saturation below 95%).

### US study site: exercise-induced dehydration study recruitment and enrollment

We recruited at the R3VIVE Fitness in Boston Massachusetts 20 healthy human subject volunteers, 13 male and 7 female, no smokers, ages 22–37, and BMI 22 to 33 (see Supplemental Material). Participants were randomly assigned to treatment and non-treatment groups by choosing between blank envelopes in which their identity as Active or Control was identified. Each participant exercised in two phases of 30 min each. After the first 30 min participants exhaled aerosol was measured a second time. Those participants in the active group received by nasal inhalation the calcium-rich hypertonic salt solution via a second hand-held, mechanical-pump spray and with a median volume droplet diameter of 8–12 μm (Supplemental Material). At the end of the 60 min of exercise the exhaled aerosol of all participants was assessed a final time. Body weight was measured for all of the subjects at multiple intervals without change of clothing. Body weight was measured using a using an InBody H20N Smart Full Body Composition Analyzer Scale with an accuracy of 0.2 lbs. Oxygen saturation was measured for several of the subjects at the t = 0, 30 min and 60 min, and several of the subjects also exhaled aerosol at t = 0 with a residual volume breathing maneuver. Pulse oxygen saturation pressure was measured using a Massimo pulse oximeter (Mighty Sat). We obtained an exemption from IRB approval from E&I Review. All participants provided written informed consent prior to enrollment. Six subjects repeated maneuvers on two separate days, and 4 subjects were excluded from the study on one of the two days of the study either for having consumed water during the study or starting a breathing maneuver without allowing time to recover normal tidal breathing—forced (residual-volume) breathing leading to an amplification of respiratory droplet generation as further discussed in the Supplemental Material.

### Exhaled aerosol assessment

We measured exhaled particles in two independent ways in our three studies in the US, Germany and India. In the US and India studies we used a *Non-Dried Droplet Counter Method*—which has the advantage of assessing the actual droplet sizes as exhaled while the disadvantage of exhaled droplet size depending on atmospheric conditions. Exhaled particles were measured before and after administration of the active or the control by a particle detector (Climet 450-t) designed to count airborne particles in the size range of 0.3 μm to greater than 5 μm. The particle detector air port was attached by a flexible plastic tube to the side (by a T connector) of a 1″ inner diameter tube into which subjects inhaled and exhaled. The 1″ tube connected at one end a mouthpiece provided with standard nebulizer tubing and at the other end a portable HEPA filter. The entire tubing system facilitated the filtration of all environmental particles from the lungs of subjects over a period of about one minute of breathing with subjects’ lips tightly sealed around the mouthpiece and pinching their noses. The rate of flow of the particle counter (50 L/min) was near the typical peak inspiratory/expiratory rate of flow of human subject breathing such that the direction of air flow remained into the particle counter. Each standard nebulizer tubing and mouthpiece were removed from sealed packaging before each subject prior to the subject’s first exhaled particle detection. On subsequent counting procedures, the same mouthpiece, tubing and HEPA filter were reattached by the participant to insure the absence of contamination from one subject to the next. Before each test, the mouthpiece was replaced by a stopper and the particle detector was turned on to verify the absence of leakage of particles from the environment. Background of less than 10 particles per liter of air was deemed “well sealed”. With the mouthpiece placed back onto the tubing, subjects performed normal tidal breathing through the mouthpiece while plugging their noses with their fingers over 1 to 2 min—beginning with two deep breaths to empty their lungs of environmental particles. Over this time frame particle counts per liter of air pulled from the exhaled breath into the particle counter diminished and subsequently fluctuated around a baseline number. Given the assurance of no leakage from the outside environment, the tight lip seal and the pinched nose, we assumed this baseline number to equate to the particles generated within the subject’s airways. Once the lower plateau of particle counts was reached subjects continued to breathe normally for the determination of exhaled aerosol particle number. Participants sat opposite to the study administrator with a plexiglass barrier in between. The Climet 450-t particle counter reports particle counts as a function of aerodynamic particle size ranges for particles larger than 0.3 μm, particles larger than 0.5 μm, particles larger than 1 μm, and all particles larger than 5 μm. The numbers reported represent average values of particle counts automatically measured by the light-scattering detector over six seconds. For our determination of exhaled aerosol particle number we averaged three to eight average particle counts (each integrating a six second interval) as reported by the particle detector to determine the mean exhaled particle count and the standard deviation. In the German studies we used a *Dried Droplet Counter Method*. This approach has the advantage of controlling humidity variability in the drying of exhaled droplets by shrinking exhaled droplets in the process of counting—while it is an underestimate of the actual droplet sizes exhaled. Exhaled particles are counted and sized in a similar way as in the Non-Dried Method, while involving drying of exhaled droplets and assessment of droplets as small as 150 nm. The Dried Droplet Counter method involved an aerosol spectrometer (Resp-Aer-Meter, Palas GmbH, Karlsruhe, Germany), specifically designed to detect airborne exhaled particles in the size range of 0.15–5.0 μm with very high sizing resolution (16 channels/decade). The optical sensor utilized a polychromatic light source to create a well-defined optical measurement volume, with every particle traveling through the measured volume generating a scattered light pulse. The size and quantity of particles were determined from the number and intensity of the scattered light pulses. Given the lower flow-rate through the system relative to the US and India measurement system (for the German system the flow rate was 9.5 L/min), the instrument comprised a heated hose section upstream of the measurement cell to avoid condensation effects and enable evaporation of larger droplets. The temperature and relative humidity in the sampled air was measured. Exhaled breath from subjects was collected as with the Non-Dried Droplet Counter by a t-adapter with HEPA filter, mouthpiece and connection port to the Resp-Aer-Meter via a hose. Again, to ensure effective hygiene, each sampling kit was removed from sterile packaging before each exhalation maneuver was initiated. Patients and healthy controls performed quiet tidal breathing through the mouthpiece while the nose was closed via a nose clip. The measurement lasting 1–1.5 min to determine the quantity of particles emitted from the lungs. The results of the test were directly displayed as a graphical curve (Fig. 1C), enabling calculation of the mean exhaled particle count per liter, particle size distribution, and mean particle size (in µm).

### Airway hydration delivery

We delivered the hypertonic calcium-rich salts to the upper airways with two different hand-held aerosol generators in our US and India studies. Both (see Supplemental Material) yielded salt mist droplets with a median diameter in the 8–12 μm range. Each delivered per 4 s actuation approximately 25 mg of hypertonic salt solution. Two deep nasal inhalations of approximately 4 s constituted an administration with each of the aerosol generators.

### Statistical analysis

All error bars represent 95% confidence intervals based on standard deviation values. Significance of differences in individual and collective aerosol numbers were determined by twin-tailed T Test. We calculated statistical significance of differences using a multiway analysis of variance (ANOVA) test for each set of variables. This allowed for evaluating the influence of multiple factors on the mean within a 95% confidence interval. P-values were calculated for each unique set of variables compared to baseline values. Each P-value below 0.05 was considered to be statistically different.

## Supplementary Information

Supplementary Information.

## References

[1] Mallah SL, Ghorab OK, Al-Salmi S (2021). COVID-19: Breaking down a global health crisis. Ann. Clin. Microbiol. Antimicrob..

[2] Josephson A, Kilic T, Michler JD (2021). Socioeconomic impacts of COVID-19 in low-income countries. Nat. Hum. Behav..

[3] De Pue S, Gillebert C, Dierckx E (2021). The impact of the COVID-19 pandemic on wellbeing and cognitive functioning of older adults. Sci. Rep..

[4] Trabelsi K, Ammar A, Masmoudi L, Boukhris O, Chtourou H, Bouaziz B, Brach M, Bentlage E, How D, Ahmed M, Mueller P (2021). Sleep quality and physical activity as predictors of mental wellbeing variance in older adults during COVID-19 lockdown: ECLB COVID-19 international online survey. Int. J. Environ. Res. Public Health.

[5] Mallapaty S, Callaway E, Kozlov M, Ledford H, Pickrell J, Van Noorden R (2021). How COVID vaccines shaped 2021 in eight powerful charts. Nature.

[6] Sriram K, Ÿkan Duygu Merve ÃA, Josua J, Aileen F, Beate C, Gisela C, Julius L, Stephan L, Linda B (2021). Beyond vaccines: Clinical status of prospective COVID-19 therapeutics. Front. Immunol..

[7] Jensen N, Kelly AH, Avendano M (2021). The COVID-19 pandemic underscores the need for an equity-focused global health agenda. Hum. Soc. Sci. Commun..

[8] Troeger C, Blacker BF, Khalil IA (2018). Respiratory disease number 1 killer Estimates of the global, regional, and national morbidity, mortality, and aetiologies of lower respiratory infections in 195 countries, 1990–2016: A systematic analysis for the Global Burden of Disease Study 2016. Lancet.

[9] Edwards DA, Norden B, Karnath L, Yaghi O, Roy CJ, Johanson D, Ott M, Brownstein J, Grove J, Tomson G, Friberg P (2021). Hydration for clean air today. Mol. Front. J..

[10] Ghosh A, Boucher RC, Tarran R (2015). Airway hydration and COPD. Cell Mol. Life Sci..

[11] Moriyama M, Hugentobler WJ, Iwasaki A (2021). Seasonality of respiratory infections. Annu. Rev. Virol..

[12] Lauc G, Markotić A, Gornik I, Primorac D (2020). Fighting COVID-19 with water. J. Glob. Health.

[13] Chumlea WC, Guo SS, Zeller CM, Reo NV, Siervogel RM (1999). Total body water data for white adults 18 to 64 years of age: The Fels Longitudinal Study. Kidney Int..

[14] Ritz P, Vol S, Berrut G, Tack I, Arnaud MJ, Tichet J (2008). Influence of gender and body composition on hydration and body water spaces. Clin. Nutr..

[15] Stookey JD, Allu PKR, Chabas D, Pearce D, Lang F (2020). Hypotheses about sub-optimal hydration in the weeks *before* coronavirus disease (COVID-19) as a risk factor for dying from COVID-19. Med. Hypotheses..

[16] Fronius M, Clauss WG, Althaus M (2012). Why do we have to move fluid to be able to breathe. Front. Physiol..

[17] Hou YJ (2020). SARS-CoV2 reverse genetics reveals a variable infection gradient in the respiratory tract. Cell.

[18] Zieliński J, Przybylski J (2012). How much water is lost during breathing?. Pneumonol. Alergol. Pol..

[19] D’Amato M, Molino A, Calabrese G, Cecchi L, Annesi-Maesano I, D’Amato G (2018). The impact of cold on the respiratory tract and its consequences to respiratory health. Clin. Transl. Allergy.

[20] Kudo E, Song E, Yockey LJ, Rakib T, Wong PW (2019). Low ambient humidity impairs barrier function and innate resistance against influenza infection. PNAS.

[21] Barbet JP, Chauveau M, Labbe S, Lockhart A (1988). Breathing dry air causes acute epithelial damage and inflammation of the guinea pig trachea. J. Appl. Physiol..

[22] Wolkoff P (2018). The mystery of dry indoor air: An overview. Environ. Int..

[23] Romaszko-Wojtowicz A, Cymes I, Dragańska E (2020). Relationship between biometeorological factors and the number of hospitalizations due to asthma. Sci. Rep..

[24] Kudo E, Song E, Yockey LJ, Rakib T, Wong PW, Homer RJ, Iwasaki A (2019). Influenza worsens dry air Low ambient humidity impairs barrier function and innate resistance against influenza infection. Proc. Natl. Acad. Sci. USA.

[25] Mecenas P, Bastos R, Vallinoto A, Normando D (2020). Effects of temperature and humidity on the spread of COVID-19: A systematic review. PLoS ONE.

[26] Rosen C, Simpson C (2008). Operative Techniques in Laryngology.

[27] Finck C, Lejeune L, Brudzynski SM (2010). Structure and oscillatory function of the vocal folds. Handbook of Behavioral Neuroscience.

[28] Zhuang P, Swinarska JT, Robieux CF, Hoffman HR, Lin S, Jiang JJ (2013). Measurement of phonation threshold power in normal and disordered voice production. Ann. Otol. Rhinol. Laryngol..

[29] Finklehor BK, Titze IR, Durham PL (1988). The effect of viscosity changes in the vocal folds on the range of oscillation. J. Voice.

[30] Sasaki C, Weaver M (1997). Physiology of the larynx. Am. J. Med..

[31] Scheinherr, A. *Glottal Motion and Its Impact on Airflow and Aerosol Deposition in Upper Airways During Human Breathing*. PhD Thesis, Fluids mechanics/Physics. Ecole Centrale Marseille (2015).

[32] Sivasankar M, Leyden C (2010). The role of hydration in vocal fold physiology. Curr. Opin. Otolaryngol. Head Neck Surg..

[33] Van Hirtum A, Bouvet A, Pelorson X (2018). Pressure drop for adiabatic air-water flow through a time-varying constriction. Phys Fluids.

[34] Bouvet A, Pelorson X, van Hirtum A (2020). Influence of water spraying on an oscillating channel. J. Fluids Struct..

[35] Xi J, Longest W, Martonin T (2008). Effects of the laryngeal jet on nano- and microparticle transport and deposition in an approximate model of the upper tracheobronchial airways. J. Appl Phys..

[36] Xi J, Si A, Dong H, Zhong H (2018). Effects of glottis motion on airflow and energy expenditure in a human upper airway model. Eur. J. Mech. B.

[37] Peng C-A, Jurman L, McCready M (1991). Formation of solitary waves on gas-sheared liquid layers. Int. J. Multiph. Flow.

[38] Watanabe W, Why W (2007). inhaling salt water changes what we exhale. J. Colloid Interface Sci..

[39] Stadnytskyi V, Anfinruud P, Bax A (2021). Breathing, speaking, coughing or sneezing: What drives transmission of SARS-CoV-2?. J. Int. Med..

[40] Scheuch G (2020). Breathing is enough: For the spread of influenza virus and SARS-CoV-2 by breathing only. J. Aerosol Med. Pulm. Drug Deliv..

[41] Field R, Moellis N, Salzman J, Bax A, Ausiello D, Woodword W, Wu X, Domici F, Edwards D (2021). Moisture and airborne salt suppress respiratory droplet generation and may reduce COVID-19 incidence and death. Mol. Front. J..

[42] Calmet H (2019). Nasal sprayed particle deposition in a human nasal cavity under different inhalation conditions. PLoS ONE.

[43] Alves M, Kruger E, Pillay B, van Lierde K, van der Linde J (2019). The effect of hydration on voice quality in adults: A systematic review. J. Voice.

[44] Edwards DA (2004). Inhaling to mitigate exhaled bioaerosols. Proc. Natl. Acad. Sci. USA.

[45] Hamed R, Schenck DM, Fiegel J (2020). Surface rheological properties alter aerosol formation from mucus mimetic surfaces. Soft Matter.

[46] Crowther RS, Marriott C (1984). Counter-ion binding to mucus glycoproteins. J. Pharm. Pharmacol..

[47] Edwards DA, Hickey A, Batycky R, Griel L, Lipp M, Dehaan W, Clarke R, Hava D, Perry J, Laurenzi B, Curran AK (2020). A new natural defense against airborne pathogens. QRB Discov..

[48] Edwards DA, Salzman J, Devlin T, Langer R (2020). Nasal calcium-rich salts for cleaning airborne particles from the airways of essential workers, students, and a family in quarantine. Mol. Front. J..

[49] George CE, Salzman J, Inbaraj LR, Chandrasingh S, Klein C, Morawska L, Edwards DA (2020). Airway hygiene in children and adults for lowering respiratory droplet exposure in health and learning environments in clean and dirty air. Mol. Front. J..

[50] Vishnoy, A. *Insacog Data Too Confirm Rise of B.1.617 Across States*. (India Economic Times, 2021). https://economictimes.indiatimes.com/news/india/insacog-data-too-confirm-rise-of-b-1-617-across-states/articleshow/82781103.cms?utm_source=contentofinterest&utm_medium=text&utm_campaign=cppst.

[51] Edwards DA, Ausiello D, Salzman J, Devlin T, Langer R, Beddingfield B, Dears AC, Doyle-Meyers LA, Redmann RK, Killeen SZ, Maness NJ, Roy CJ (2021). Exhaled aerosol increases with COVID-19 infection, age, and obesity. PNAS.

[52] Bake B, Larsson P, Ljungkvist G (2019). Exhaled particles and small airways. Respir Res.

[53] Song D, Chan D, Duncan GA (2020). Mucin biopolymers and their barrier function at airway surfaces. Langmuir.

[54] Etzold M, Linden P, Worster M (2021). Transpiration through hydrogels. J. Fluid Mech..

[55] Vyazmin A, Khramtsov D, Pokusaev B, Nekrasov D, Zakharov N (2019). Experimental study of liquid evaporation from a surface of gel mixture. EPJ Web Conf..

[56] Singanayagam A, Hakki S, Dunning J, Madon KJ, Crone MA, Koycheva A (2021). Community transmission and viral load kinetics of the SARS-CoV-2 delta (B.1.617.2) variant in vaccinated and unvaccinated individuals in the UK: A prospective, longitudinal, cohort study. The Lancet.

[57] Callaway E (2021). Delta coronavirus variant: Scientists brace for impact. Nature.

[58] Greenhalgh T (2021). Ten scientific reasons in support of airborne transmission of SARS-CoV-2. The Lanccet.

[59] Vanderlei FM (2013). Effects of different protocols of hydration on cardiorespiratory parameters during exercise and recovery. Int. Arch. Med..

