# Supplementary material for: COVID-19 symptoms are reduced by targeted hydration of the nose, larynx and trachea
Source: Sci Rep. 2022 Mar 29;12:4599. doi: 10.1038/s41598-022-08609-y (PMC8964810; doi:10.1038/s41598-022-08609-y)
Supplement: Supplementary file 1 — Supplementary Information. [file 41598_2022_8609_MOESM1_ESM.docx]

**Supplemental Material**

**COVID-19 symptoms are reduced by targeted hydration of the nose, larynx and trachea**Carolin Elizabeth George,^1*^ Gerhard Scheuch,^2*^Ulf Seifart,^3^ Leeberk Raja Inbaraj,^1^ Sindhulina Chandrasingh,^1^ Indu K Nair,^1^ Anthony J. Hickey,^4^ Michael R. Barer,^5^ Eve Fletcher,^5^ Rachel Field,^6^  Jonathan Salzman^7^, Nathan Moelis,^8^ Dennis Ausiello^9^ and David A. Edwards^7,10*^

^1^Bangalore Baptist Hospital, Bangalore India

^2^GS BIO-INHALATION GmbH, Germunden, Germany

^3^Klinik Sonnenblick, Marburg Germany

^4^RTI International, Research Triangle Park, NC

^5^Respiratory Sciences, University of Leicester, UK

^6^School of Bioengineering, Columbia University, New York, New York

^7^Sensory Cloud, 650 East Kendall St, Cambridge MA

^8^School of Bioengineering, Northeastern University, Huntington Avenue

^9^Harvard Medical School, Boston MA

^10^School of Engineering & Applied Sciences, Harvard University, Cambridge MA

*Corresponding authors

1. **Marburg Germany Study Participant Data**
2. **Boston US Study Participant Data**
3. **Bangalore India Data & Random Control Trial Sample Size**
4. **FEND Mister Delivery Systems**
5. **Tidal versus Residual Volume Breathing Exhaled Aerosol**
6. **Assessment of SARS-CoV-2 RNA in Exhaled Breath**
7. **Nasal Saline, Post-Nasal Drip & Non-Treatment Control Results**
8. **Marburg Germany Study Participant Data**

We recruited 357 healthy human subject volunteers at the University Marburg from January to March 2021. Of these there were 212 male and 145 female, 65 smokers and 292 non-smokers, ages 18-83, and BMI 17 to 43. Find blinded personal and exhaled aerosol data on the 357 subjects [here](https://drive.google.com/drive/folders/1K6P-qTPEP5FewSC2ieZ8Zq7kKCp5Hu99?usp=sharing) (https://drive.google.com/drive/folders/1K6P-qTPEP5FewSC2ieZ8Zq7kKCp5Hu99).

1. **Boston US Study Participant Data**

We recruited at the R3VIVE Fitness in Boston Massachusetts 20 healthy human subject volunteers, 13 male and 7 female, no smokers, ages 22-37, and BMI 22 to 33. Find blinded personal and exhaled aerosol data on the 357 subjects [here](https://drive.google.com/drive/folders/1BLEFJC99CIgWVZ-Hzudgr5F6A_c3pbF2?usp=sharing) (https://drive.google.com/drive/folders/1BLEFJC99CIgWVZ-Hzudgr5F6A_c3pbF2?usp=sharing).

3. **Bangalore India Data & Random Control Trial Sample Size**

We recruited 87 human volunteers between December 2020 and June 2021. Find blinded personal data [here](https://drive.google.com/drive/folders/1XmOrtg7FGV9AFIGnIFwxDTmUdPw7VMIQ?usp=sharing) (https://drive.google.com/drive/folders/1XmOrtg7FGV9AFIGnIFwxDTmUdPw7VMIQ?usp=sharing). We measured their exhaled aerosol prior to treatment with FEND or Simply Saline and for the majority of volunteers post FEND or Simply Saline treatment. Find blinded exhaled aerosol data [here](https://docs.google.com/file/d/1gbLGn4832J9abkyd4aEYhJiqSDAwPW-o/edit?usp=docslist_api&filetype=msexcel) (https://docs.google.com/file/d/1gbLGn4832J9abkyd4aEYhJiqSDAwPW-o/edit?usp=docslist_api&filetype=msexcel).

We powered our randomized control study based on a sample size estimate using a statistical power analysis for two independent variables with continuous outcome and using exhaled aerosol as primary outcome variable. In the analysis we used a 95% confidence margin (Z=1.96), a target margin of error of 20 to 25%, and a standard deviation of 33%. We used the outcome data from our previous Bangalore Baptist Hospital study of exhaled aerosol (39) with 20 human volunteers in the active group to estimate the necessary size of the active group in the present study. Sample size estimate ranged from 14 to 22 based on an error margin of 25% or 20% respectively. These results led us to choose a sample size of 20 active and 20 control for our random control study.

4. **FEND Mister Delivery Systems**

We used two different aerosol generators for delivering the calcium-rich hypertonic saline (FEND) solutions to the upper airways (Fig S1). Both delivered similar droplet sizes by a similar maneuver of two deep nasal inhalations of the mist once generated before the nose. A pocket-size pump-spray device (Fig S1A) functioned with a mechanical aerosol generator (AeroPump, Germany) by pressing the FEND solution through laser-etched holes in a silica wafer followed by Rayleigh-Taylor instability that disintegrates into droplets of size distribution shown in Fig S2A. A table-top nebulizer device (Fig S1B) functioned with a vibrating mesh on tilting the device promoting the generation of droplets of size distribution shown in Fig S2B. Emitted size distributions from the two FEND delivery devices and the Simply Saline pump spray (Fig S2B) were determined via laser diffraction using a Spraytec spray analysis system (Malvern Panlytical Ltd, UK) in an open-bench configuration. The delivery devices were affixed approximately 2” from the measurement beam. Data collection occurred at a 1 kHz acquisition rate over the duration of the spray event, with reported results representing the time-averaged size distributions. All experimental conditions were assessed in triplicate.

5. **Tidal versus Residual Volume Breathing Exhaled Aerosol**

To assess the effect of breathing maneuver on exhaled aerosol particle number we evaluated exhaled aerosol using the non-dried method described in the article with six of the volunteers from the Boston study (subjects 2, 9, 13, 18, 19, 20). Each subject was coached to breathe into the detection system with either an easy tidal breathing (TB) maneuver or a forced exhalation maneuver aimed to empty the lungs of residual volume (RV) air. Exhaled aerosol for the RV maneuver significantly exceeded exhaled aerosol for the TB maneuver for 5 of the 6 subjects (P<0.002). Subject 13 exhaled the same relatively high number of particles (P=0.56) on both maneuvers, reflecting either deep breathing on both maneuvers or a respiratory droplet generation independent of breathing type. All of the subjects subsequently performed the exercise routine of the Boston study (the second of two experiments on separate days by subjects 2 and 9), while at 30 minutes three of the subjects (2, 9, 20) received the upper airway calcium-rich hypertonic salt active treatment. At the conclusion of the 60 minute workout all subjects breathed heavily with exhaled aerosol numbers elevated as indicated in the RV maneuver (Fig S3). Exhaled aerosol for subjects 2, 9 and 20 however fell subsequently relative to their 30 minute exhaled aerosol number, indicating a suppression of exhaled aerosol on the TB maneuver (30 minutes following the upper airway hypertonic salt delivery), while not on the RB maneuver. While preliminary, these results indicate that the RV maneuver may tend to generate many more particles from a location beyond the upper airways, possibly in the small airways through closing off of airways as suggested in Fig 1C and, indeed, promoted by the residual volume maneuver itself.

6. **Assessment of SARS-CoV-2 RNA in Exhaled Breath**

We used a face-mask sampling (FMS) described elsewhere (27) in an attempt to detect and quantify exhaled SARS-CoV-2. Twenty one of the 87 subjects in our study, all detected with COVID-19 within one to seven days of first symptoms in late March 2021, wore specially-prepared face masks containing two 1×9 cm 3D printed polyvinyl-alcohol (PVA) sampling matrix strips placed horizontally across the inside of the mask for 30 min. Each patient wore two fresh masks — one before and one 2 hours after administration of FEND. Exposed masks were processed by removal, heating to 121C for one hour, following which the matrix strips were dissolved and analyzed by RT-qPCR. To evaluate the impact of the 121C heating step, a precaution taken to eliminate risk of exposure on transport of the samples while location of testing lab remained uncertain during the health crisis at Bangalore Baptist Hospital in spring 2021, we exposed masks to SARS-CoV-2 at the University of Leicester and compared viral genome copies on masks subsequently heated to 121C and not heated. Viral genome copy numbers were determined by copies per strip for FMS both of the control masks (heat treated and not) and the patient-worn samples. We determined a dramatic reduction in viral genome numbers (potentially 3-4 orders of magnitude) in the control tests for those masks heated to 121C for one hour relative to those masks not heated. In 4 of the 80 patient mask samples collected at BBH we detected low (less than 200 genome copies per strip) levels of virus. Given these results and the impact of the inactivation step we conclude that SARS-CoV-2 was present in the exhaled aerosol of the human subjects in our study.

7. **Nasal Saline, Post-Nasal Drip & Non-Treatment Control Results**

We evaluated the progression of illness among 10 of the 87 subjects not included in the randomized control study and not treated with either FEND or Simply saline (see Table S1). Exhaled aerosol for the untreated group is shown in Figure S4. No diminution of exhaled aerosol can be seen over time, similar to the control group in the study (Figure 4B), while without any of the subjects showing a trend to lower exhaled aerosol, unlike the Simply Saline control. Three of the 10 subjects had high CRP levels, all of whom required IV antibiotic intervention. Oxygen saturation levels did not change significantly for the group over the course of hospitalization nor was there significant change in symptom scores over the course of hospitalization (see Figure S2). Comparison of these results with the results of the randomized controlled study, and notably comparison of Figure S4 with Figure 4B), suggests the possibility of some efficacy of the Simply Saline treatment, possibly owing to post-nasal drip occurring in the bed-ridden subjects.

**Table S1.** Blinded personal data for the 10 (of 87) mildly symptomatic covid-19 subjects at Bangalore Baptist for whom longitudinal data were collected from admission to discharge without treatment (active or control).


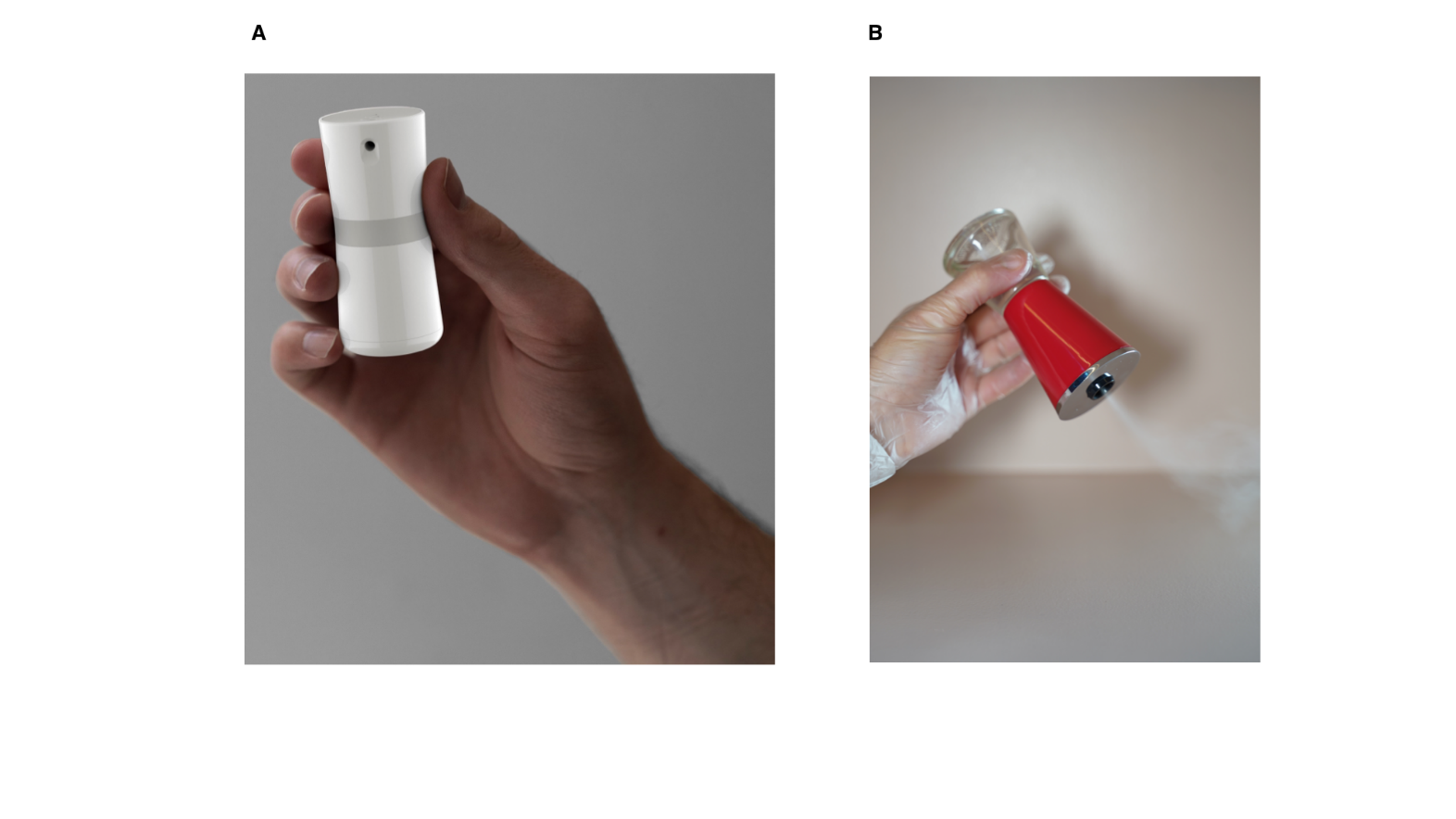


**b**

**a**

**Figure S1**. Two aerosol generation devices used to deliver the calcium-rich hypertonic saline (FEND) in the human volunteer studies. (a) A pocket-size pump-spray device; and (b) A table-top nebulizer device. Both devices were manufactured by Sensory Cloud Inc.

**b**

**a**


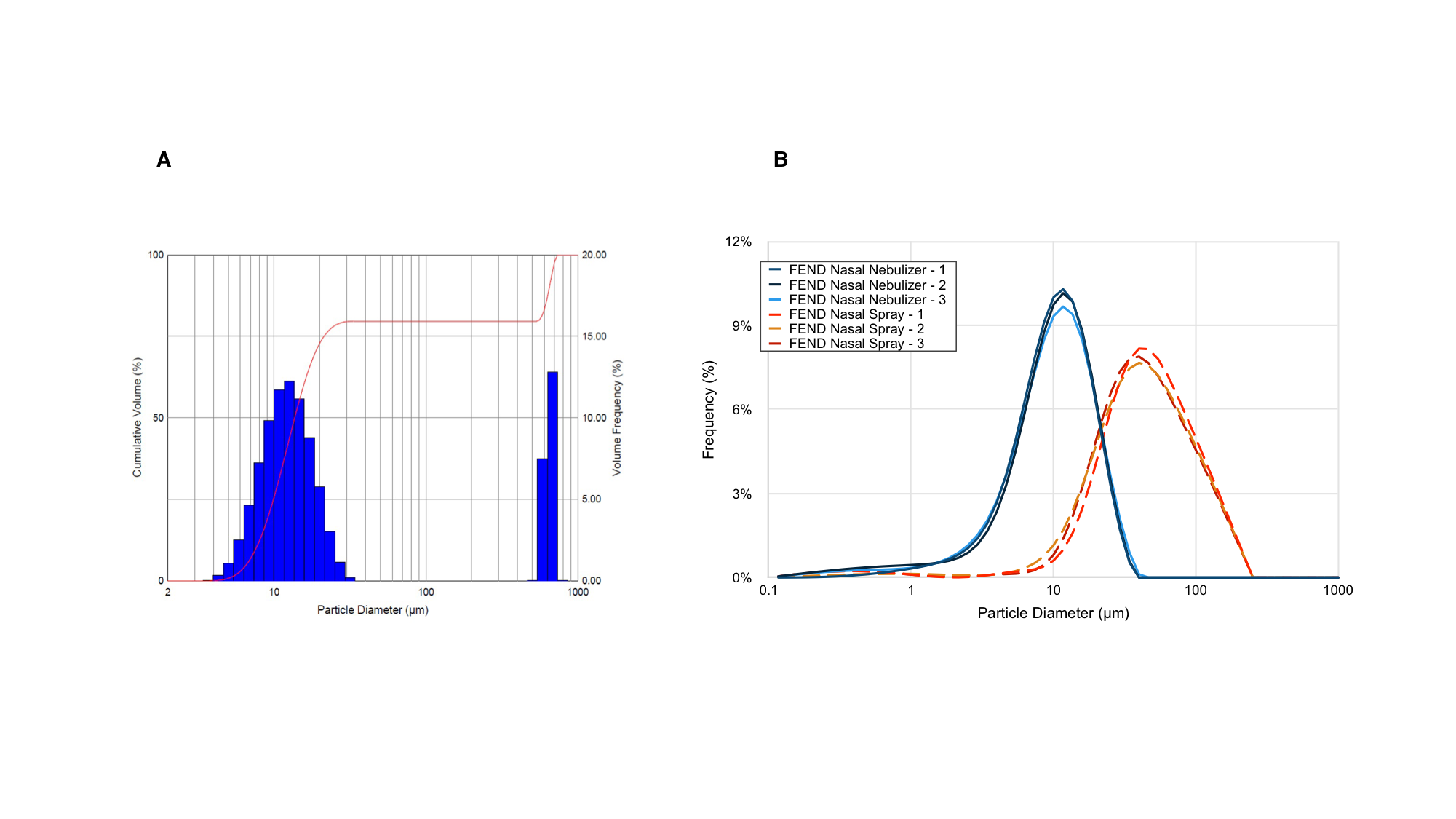

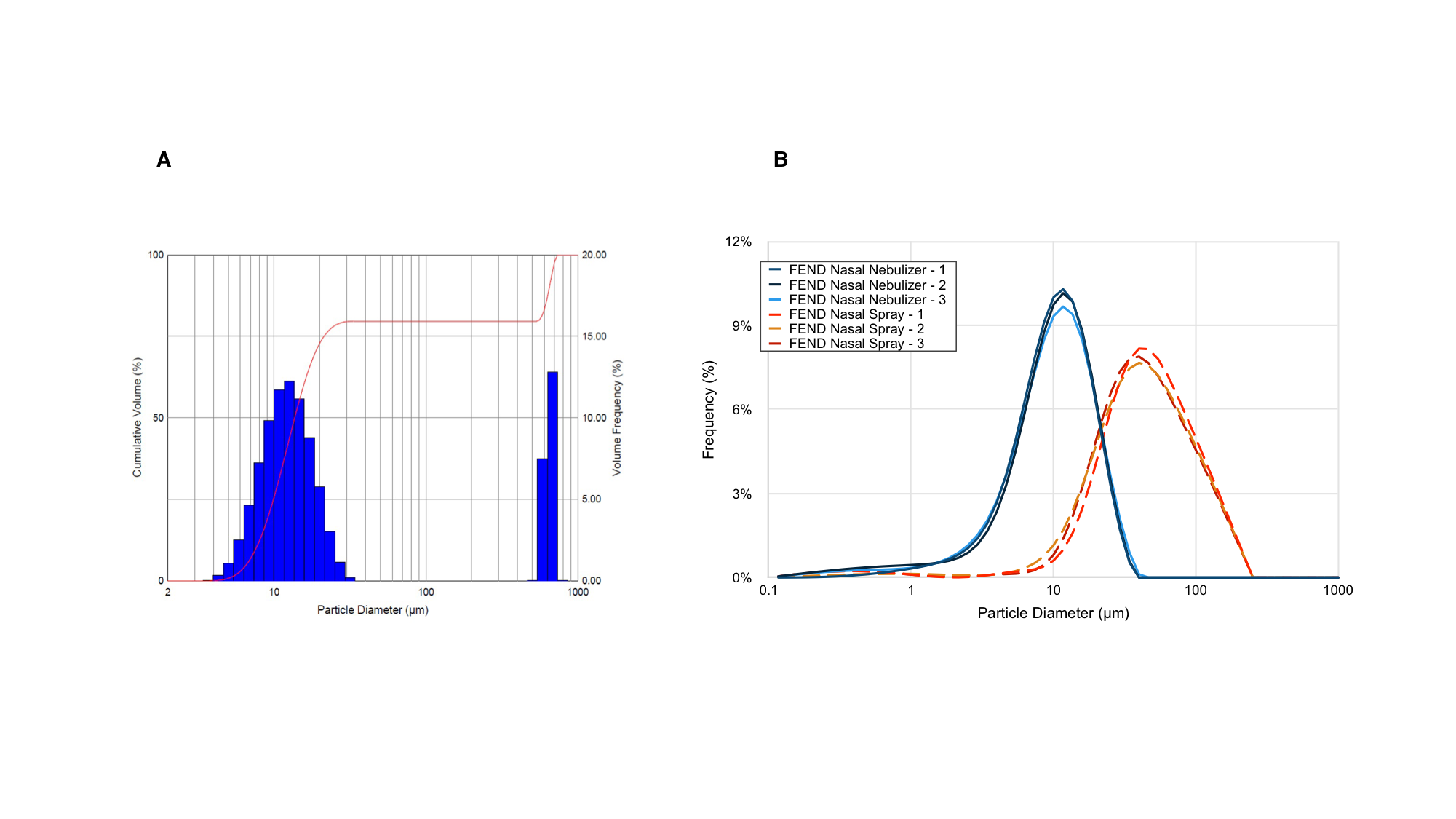


Figure S2. Particle size distributions from the two aerosol generation devices used in the study for delivery of the active as well as the spray device used for the control. An open-beam laser diffraction system (Malvern Spraytec) assessed geometric size distributions of emitted droplets: (a) from the pocket FEND aerosolizer; and (b) from the table-top aerosolizer as well as the nasal (control) spray. The black and red distributions reflect distributions that preferentially deposit in the nose, trachea and main bronchi (black) and in the nose only (red).


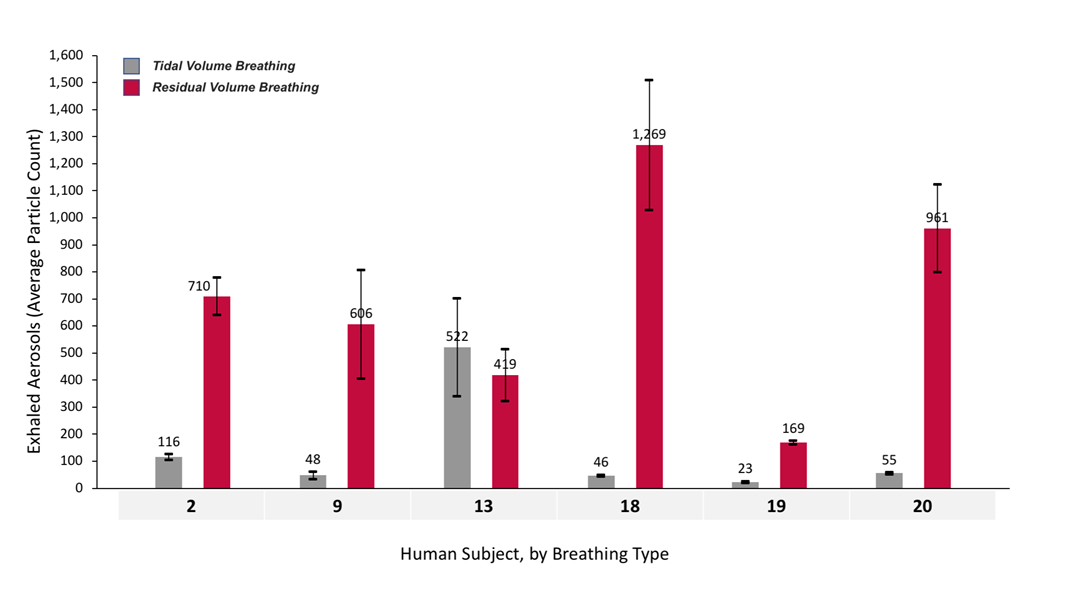


**Figure S3.** Exhaled aerosol particle numbers from 6 human subjects in the Boston study following normal tidal breathing and residual volume breathing. Error bars represent standard errors of the mean.


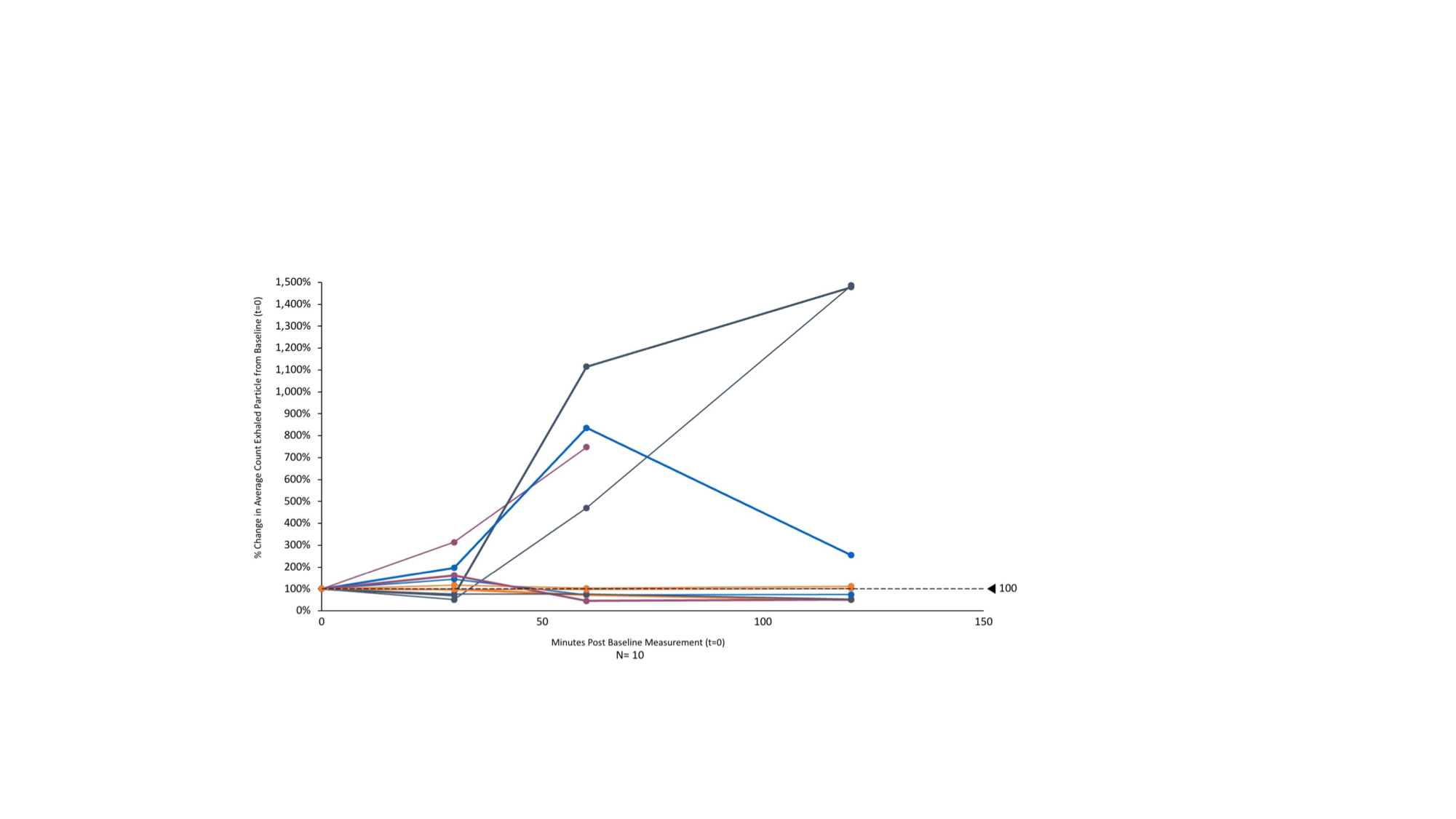


**Figure S4.** Exhaled aerosol particle numbers from the 10 non-treated mildly symptomatic covid-19 subjects at Bangalore Baptist hospital following their recent infection between December 2020 and June 2021.


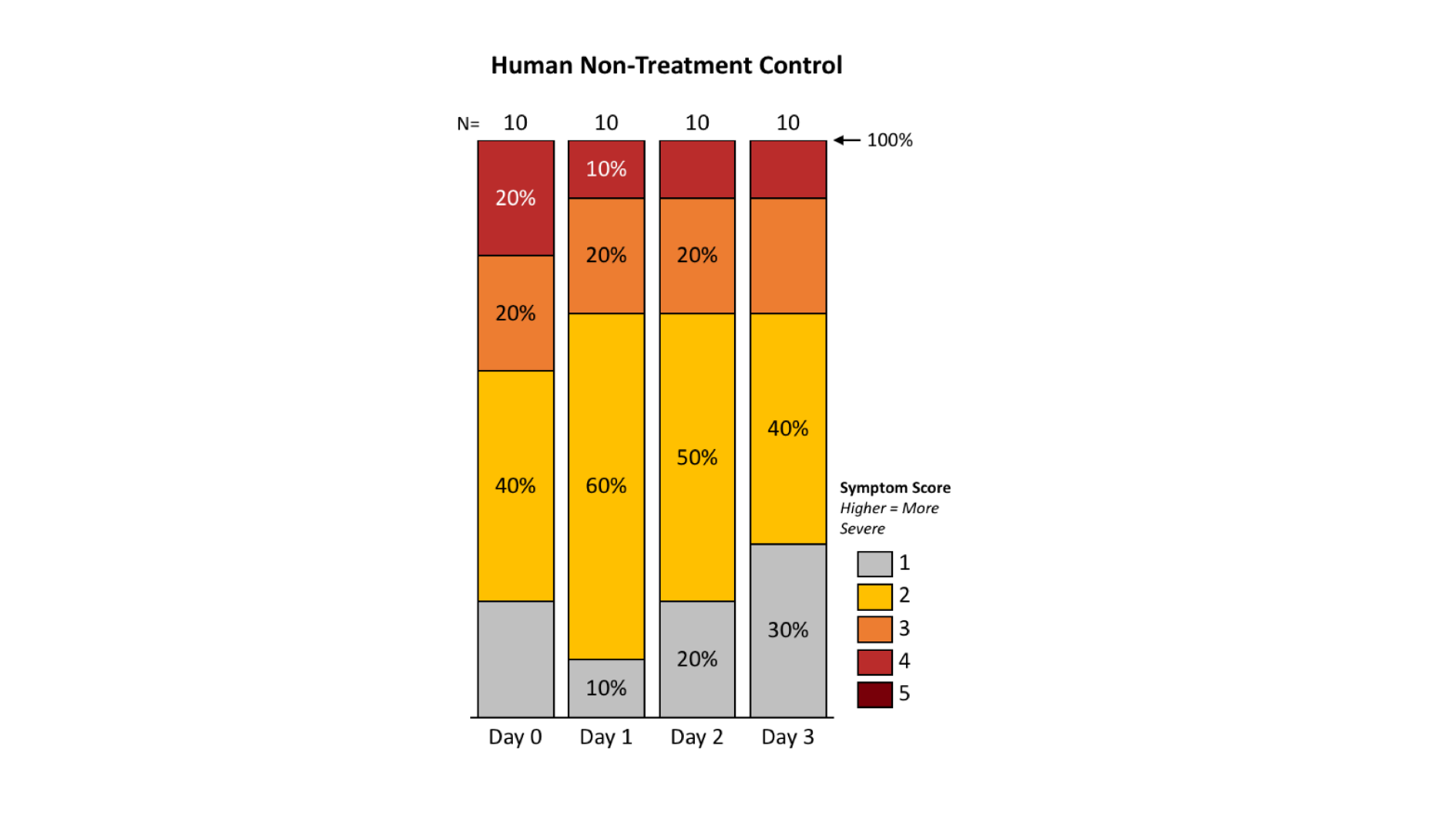


**Figure S5**. Self-reported symptom scores (on scale 1 to 5, with 1 = no symptoms, and 5 = most severe symptoms) as a function of days of hospitalization and administration over the days of hospitalization of the 10 non-treated mildly symptomatic COVID-19 patients.
